# Involve fathers in family dynamics and in early interactions with children, in the face of cultural factors

**DOI:** 10.1192/j.eurpsy.2024.240

**Published:** 2024-08-27

**Authors:** E. Dozio, I. Pueugueu

**Affiliations:** ^1^Action contre la Faim, Paris, France; ^2^Action contre la Faim, Goma, Congo, The Democratic Republic of the

## Abstract

**Introduction:**

In the first years of life, parents and a secure family environment are essential to the survival and development of young children.

Attention is focused on the undeniable importance of mothers’ role in childcare. But it’s also important to involve fathers, who are often sidelined from the responsibilities of this role, not least because of cultural factors linked to the separation of roles. In some situations, this is compounded by the psychological suffering that men may feel, without being able to admit it or express it, as a result of representations linked to masculinity.

**Objectives:**

The aim of the intervention was to strengthen the psychosocial and parenting skills of men, while taking into account their distress. The objective was to reduce intra-family violence, to involve men more in family life and in the care of young children, and to work on cultural representations of the role and cultural dynamics within the family and the community.

**Methods:**

Men, fathers and future fathers were recruited in the Mweso region in the Democratic Republic of Congo, following community psychoeducation. The group protocol took the form of five weekly sessions covering various themes linked to psychological distress, emotion management, psychosocial skills as well as gender roles and child development.

**Results:**

Between 2021 and 2023, 727 men participated in the program. They showed an improvement in well-being (reduction in anger, symptoms of anxiety, depression and PTSD), better management of emotions and the acquisition of strategies to address cultural factors linked to fatherhood within the family unit and the community.

**Conclusions:**

The use of this protocol allowed men to become more aware of the issues of psychological suffering and fatherhood linked to cultural factors by allowing them better inclusion in the family dynamic.

**Disclosure of Interest:**

None Declared

